# CNN-based ranking for biomedical entity normalization

**DOI:** 10.1186/s12859-017-1805-7

**Published:** 2017-10-03

**Authors:** Haodi Li, Qingcai Chen, Buzhou Tang, Xiaolong Wang, Hua Xu, Baohua Wang, Dong Huang

**Affiliations:** 1 Key Laboratory of Network Oriented Intelligent Computation, Harbin Institute of Technology, Shenzhen, GuangDong China; 2 Key Laboratory of Symbolic Computation and Knowledge Engineering of Ministry of Education, Jilin University, Jilin, China; 3 School of Biomedical Informatics, University of Texas Health Science Center at Houston, Houston, Texas USA; 4 College of Mathematics and statistics, Shenzhen University, Shenzhen, GuangDong China

**Keywords:** Biomedical entity normalization, Convolutional neural network

## Abstract

**Background:**

Most state-of-the-art biomedical entity normalization systems, such as rule-based systems, merely rely on morphological information of entity mentions, but rarely consider their semantic information. In this paper, we introduce a novel convolutional neural network (CNN) architecture that regards biomedical entity normalization as a ranking problem and benefits from semantic information of biomedical entities.

**Results:**

The CNN-based ranking method first generates candidates using handcrafted rules, and then ranks the candidates according to their semantic information modeled by CNN as well as their morphological information. Experiments on two benchmark datasets for biomedical entity normalization show that our proposed CNN-based ranking method outperforms traditional rule-based method with state-of-the-art performance.

**Conclusions:**

We propose a CNN architecture that regards biomedical entity normalization as a ranking problem. Comparison results show that semantic information is beneficial to biomedical entity normalization and can be well combined with morphological information in our CNN architecture for further improvement.

## Background

Entity normalization (i.e., entity linking) is a fundamental task of information extraction which aims to map entity mentions in text to standard entities in a given knowledge base (KB), and still is challenging. The main challenges of entity normalization lie in: 1) ambiguity - the same entity mention may refer to different standard entities, 2) variation - different entity mentions may refer to the same standard entity, and 3) absence - entity mentions may not be mapped to any standard entity in the given knowledge base. In the newswire domain, ambiguity is the primary challenge. In some entity types in the biomedical domain (i.e., diseases, drugs), however, variation is much more common than ambiguity, and absence occurs sometimes. Because of this, most entity normalization systems in the newswire domain focus on dealing with the disambiguation problem using machine learning methods based on context of entity mentions, while, in the biomedical domain, lots of systems focus on the variation problem and use rule-based methods relying on morphological information of entity mentions. Although the rule-based methods achieve good performance on medical entity normalization, they still suffer from several limitations. Firstly, it is impossible to collect complete rules to cover all mentions of a type of entity as their descriptions are various and changing. Secondly, rules for one type of entities are not directly applicable to another type of entities. For example, synonym dictionary of disorders obviously cannot be applicable to medications. Thirdly, it is difficult to handle the following two cases: 1) mentions with similar morphology but totally different meaning such as “ADA-SCID (adenosine deaminase deficiency)” and “X-SCID (X-linked combined immunodeficiency diseases”; and 2) mentions with significant different morphology but similar semantic meanings such as “kaplan plauchu fitch syndrome” and “acrocraniofacial dysostosis”.

The goal of this paper is to advance biomedical entity normalization by utilizing semantic information of biomedical entity mentions. To achieve this goal, we design a CNN architecture to capture semantic information of biomedical entity mentions used for ranking candidates generated by handcrafted rules used in traditional rule-based systems. In order to tackle the absence problem, we add ‘NIL’ (denotes absence) into the candidate set of an entity mention when necessary. Then, we can handle variation and absence in a unified framework.

Our main contributions are: 1) we propose a novel CNN-based ranking method for biomedical entity normalization, which takes advantages of CNN in modeling semantic similarities of entity mentions; 2) our system achieves state-of-the-art results on two benchmark datasets.

### Related work

In last decades, a large number of studies have been proposed for biomedical entity normalization, however, nearly all of them focus on how to use morphological information to normalize entity mentions more accurately. In this section, we only discuss three state-of-the-art representative systems on the two benchmark datasets used for evaluation. Moreover, related studies using CNN for semantic matching are also briefly introduced.

UWM, the best system for the disease and disorder mention normalization task of the SemEval 2014 challenge by Ghiasvand and Kate [1] which used an expanded dataset of the eHealth task of the ShARe/CLEF 2013 challenge [2], achieved an accuracy of 89.50% on the dataset of the SheARe/ClEF 2013 challenge. It first automatically learned patterns of variations of clinical terms from the unified medical language system (UMLS)[3] and the training set of the challenge by computing edit distances between the variations, and then attempted to normalize unseen entity mentions by performing exact match between their variations generated by the learnt patterns and an entity mention in the training set or an entity in the given KB. DNorm [4] proposed by Leaman et al. adopted a pairwise learning-to-rank approach and achieved a good result on the NCBI disease dataset [5]. It adopted vector space model to represent medical entity mentions, and used a similarity matrix to measure how similar are given medical entity mentions and standard entity mentions. The similar system developed by Zhang et al. [6] were submitted to the SemEval 2014 challenge and achieved the best normalization result.

D’Souza & Ng’system (2015) [7], the best rule-based system up to date on the ShARe/CLEF datasets, was a multi-pass sieve system based on manual rules. It defined 10 kinds of rules at different priority levels to measure morphological similarities between entity mentions and entities in the given KB for normalization. TaggerOne [8], the best machine learning-based system up to date on the NCBI dataset, used semi-Markov models for biomedical entity recognition and normalization jointly.

Convolutional neural network(CNN) is a type of feed-forward artificial neural network that has been widely used to model semantic information of sequences in NLP such as sentences [9], short texts [10], questions and answers in question answering [11], etc, and then to handle sequence classification [12–14] and match problems [15, 16]. There are two works most similar to our study: the work proposed by Aiaksei and Alessandro for learning to rank short text pairs [17] and the work proposed by Limsopatham & Collier [18] for normalising medical concepts in social media texts, which consider medical concept normalization as a classification problem. Both of them do not consider any other information, such as morphologic information, except semantic information modeled by CNN.

## Methods

### Overview

Our system consists of two modules: 1) candidate generation: generating candidates for a given biomedical entity mention, 2) candidate ranking: ranking biomedical entity candidates using a CNN architecture. A detailed description of them is given in the following sections.

### Candidate generation

We use the same rules in [7] to generate candidates. In [7], 10 kinds of rules were used as sieves at 10 priority levels to normalize biomedical entities. A biomedical entity mention was normalized into an entity that was found by the sieve at the highest priority level. In our system, we divide those rules into three categories according to morphological similarity between entity mentions in text and entity mentions in a training set and a given KB:

1. exact-match (denoted by CI): an entity mention in text exactly matches an entity mention in the training set or a standard entity in the KB;
2. exact-match II (denoted by CII): an entity mention in text exactly matches an entity mention in the training set or a standard entity in the KB after morphological change;
3. partial-match (denoted by CIII): an entity mention in text cannot be found by the two categories of rules above, but some words of it appears in certain entity mentions in the training set or certain standard entities in the KB.

Given an entity mention *m*, there will be three candidate subsets generated by the rules in the three categories, denoted by *E*_1_, *E*_2_ and *E*_3_ respectively, and candidates in *E*_1_ are most morphological similar to the mention, candidates in *E*_2_ are second-most morphological similar to the mention and candidates in *E*_3_ are least morphological similar to the mention. Table 1 gives us examples of candidates of some biomedical entity mentions generated by rules in different categories.

**Table 1:** Examples of candidates of some biomedical entity mentions generated by the rules in the three categories

Category	Rule in [7]	Example	
		Mention	Candidate
Exact-Match(CI)	Dictionary-based	Coronary artery disease	Coronary artery disease
Exact-Match II (CII)	Abbreviation expansion	“Sch” in “Suxamethonium chloride (Sch)”	Suxamethonium chloride
	Subject Object	Weight gain	Gain weight
	Numbers replacement	Vitamin b2	Vitamin b ii
	Hyphenation	5-hydroxytriptamine	5 hydroxytriptamine
	Suffixation	Hypotensive	Hypotension
	Synonyms	Renal cell carcinoma	Renal cell cancer
	Stemming	Hypotensive	{Hypotension, Hypotensin}
	Composite	Optic and peripheral neuropathy	{Optic neuropathy, Peripheral neuropathy}
Partial-Match (CIII)	Partial match	Cardiac injury	{Cardiac arrest, Cardiac disorders, ⋯, Renal injury}

**Fig. 1:**
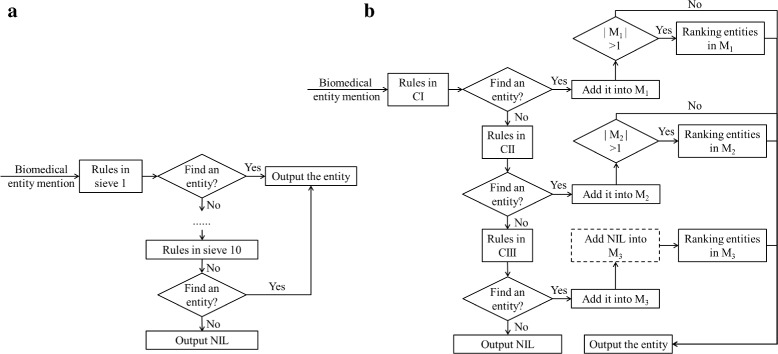
Comparison of D’Souza & Ng and ours. Two subfigure contains: (**a**) “Workflow of the system proposed by D’Souza & Ng.” and (**b**) “Workflow of our system”

In order to integrate semantic information, for an entity mention *m*, we rank all candidates in its candidate subset most morphological similar to it (denoted by *E*) according to semantic similarities between *m* and the candidates, and choose the top-ranked entity as normalization result of *m*. When there is only one element in *E* and *E* is not *M*_3_ (i.e. only one entity is found using rules in CI or CII), the element is directly chosen. When *E* is *E*_3_ and NIL is considered, we add NIL into *E* for ranking as no entity can be found using rules in CI or CII. The detailed workflow of our system is shown in Fig. 1b, which is similar to D’Souza & Ng [7] as shown in Fig. 1a.

### Candidate ranking

**Fig. 2:**
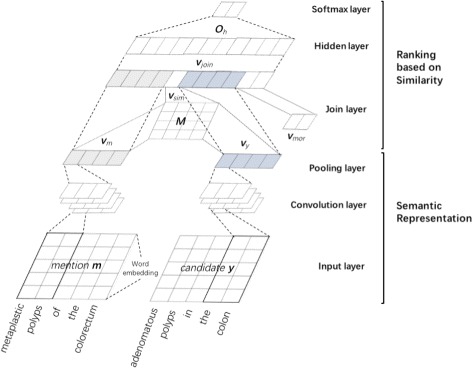
Architecture of the CNN-based ranking module

Suppose that there are *n* elements in *E*(*E*={*y*_1_,*y*_2_,⋯,*y*_*n*_}) for an entity mention *m*, we design a CNN architecture (as shown in Fig. 2) to compute similarities of mention-and-candidate pairs <*m*,*y*_1_>,<*m*,*y*_2_>,⋯,<*m*,*y*_*n*_> based on both morphological information and semantic information, and rank them. The architecture is composed of six layers, which can be divided into two parts: 1) semantic representation, including an input layer, a convolutional layer and a pooling layer; 2) ranking based on similarity, including a joint layer, a hidden layer and a soft-max layer. In our study, we use a pairwise approach in learning to rank. In the training phase, a mention-and-candidate pair <*m*,*y*_*i*_>(1≤*i*≤*n*) is labeled as 1 if it appears in the training set, otherwise, 0.

Semantic representation

In the input layer, given an entity mention *m* with *l* words and a candidate *y* with *k* words, each word *w* of an entity mention and a candidate is represented by a word embedding of size *t* (*e*_*w*_∈*R* ^*t*^). We concatenate word embeddings of all words of *m* and *y* to form their representations, a matrix of size *t*×*l* for *m* and *a* matrix of size *t*×*k* for *y*. Take *m*=*w*_1_,*w*_2_,⋯,*w*_*l*_ for example, it is represented by a matrix *x*_*m*_=[*e*_*w*1_,*e*_*w*2_,⋯,*e*_*wl*_]∈*R* ^*t*×*l*^).

In the convolutional layer, the matrix of *m* and that of *y* are converted into two groups of feature vectors by applying a convolution operator to context windows of different sizes according to filters of different sizes. Given a filter of size *c*,*s*∈*R* ^*t*×*c*^, for example, the following feature *f*_*i*_ can be generated by applying the rectified linear unit (Relu) function to a *c*-word context window of *m*, denoted by *w*_*i*:*i*+*c*−1_=*w*_*i*_ *w*_*i*+1_,⋯,*w*_*i*+*c*−1_:

$$f_{i}=Relu(s \cdot x_{i:i+c-1}+b) $$

where *x*_*i*:*i*+*c*−1_= [*e*_*wi*_,*e*_*wi*+1_,⋯,*e*_*wi*+*c*−1_] is the representation of *w*_*i*:*i*+*c*−1_ and *b*∈*R* is a bias. When applying the convolution operator with filter *s* to all possible *c*-word context windows (*i* ranging from 1 to *l*−*c*+1), we obtain a feature vector *f*= [*f*_1_,*f*_2_,⋯,*f*_*n*−*c*+1_]∈*R* ^*t*×*l*^. For different types filters of different sizes, we obtain a group of feature vectors for *m* or *y*. The number of feature vectors is the number of filters.

In the pooling layer, 1-max pooling is used to extract the most important feature from each feature vector to reduce computational complexity of the architecture. That is, $\overline {f}=max\{f_{1},f_{2},\cdots,f_{n-c+1}\}$ is extracted to represent *f* mentioned above. Suppose that there are *p* filters, we will obtain a new vector of length *p* to represent *m* or *y*, denoted by $v_{m}=[\overline {f_{1}},\overline {f_{2}}, \cdots, \overline {f_{p}}]$ for *m*, where $\overline {f_{i}}$ is the feature extracted from the *i*-th feature vector generated by the convolutional layer.

Ranking based on similarity

In the joint layer, representations of *m* and *y* (*v*_*m*_ and *v*_*y*_), semantic similarity between *m* and *y* (*v*_*sem*_) and morphological similarity vector (*v*_*mor*_) are concatenated into a single vector: *v*_*joint*_= [*v*_*m*_,*v*_*y*_,*v*_*sem*_,*v*_*mor*_], where $v_{sem}=sim(v_{m}, v_{y})= v_{m}^{T}Mv_{y}, M\in R^{p\times p}$ is a similarity matrix optimized during the training phase, and *v*_*mor*_ is a vector to represent the proportion of the same words accounting for all words in *m* and *y*, and their similarity based on word inverse mention frequency. This vector is then passed through the hidden layer, a fully connected neural network with a non-linearity activation function as follows:

$$o_{h}=Relu(r_{h} \cdot v_{joint} + {b}'), $$

where *r*_*h*_ is the weight vector of *v*_*joint*_ and *b* ^′^∈*R* is a bias. Finally, the output of the hidden layer is further fed to the softmax layer for ranking (i.e. a pairwise learning-to-rank approach). The ranking score is calculated using the following function:

$$score(m,y_{i })=\frac { e^{o^{\mathrm{T}}_{h(i)} {\mathbf q_{1 }}} }{ e^{o^{\text{T }}_{h(i)} {\mathbf q_{0 }}} + e^{o^{\mathrm{T}}_{h(i)} {\mathbf q_{1 }}} } $$

where *o*_*h*_ is the output of the hidden layer and *q*_0_ **,** *q*_1_ are weights of 0 and 1 labels, respectively. The candidate *y* that achieves highest score is chosen as the normalized entity.

### Datasets

We evaluate our approach on two benchmark datasets for biomedical entity normalization: the ShARe/CLEF eHealth dataset (ShARe/CELF)[2] and the NCBI disease dataset (NCBI)[5]. The first dataset is about disorder (i.e. disease and problem) normalization in clinical text, and the second dataset is about disease normalization in biomedical literature. They have their own reference KB, and only the Share/CLEF datset considers NIL(i.e., CUI-less). The detailed information of the three datasets is shown in Table 2, where “#*” is the number of ‘*’ such as document (doc), entity (ent), mention (men), mention identifiers (ID) and NIL.

**Table 2:** Detailed information of the three benchmark datasets used in our study

		Dataset	
		ShARe/CELF	NCBI
KB	Name	SNOMED CT	MESH & OMIM
	#ent	126525	11915
Training Set	#doc	199	692
	#men	5816	5921
	#ID	968	707
	#NIL	1638	0
Test set	#doc	99	100
	#men	5351	964
	#ID	796	201
	#NIL	1736	0

### Experimental settings

We start with a baseline system, a reimplementation of D’Souza & Ng’s system [7], investigate our CNN-based ranking system under two different settings: 1) CNN-based ranking without morphological information, and 2) CNN-based ranking with morphological information, and compare it with other state-of-the art systems, including the best challenge systems on the dataset of the ShARe/CLEF 2013 (UWM), the best rule-based systems up to date (Jennifer and Vincent’s system), and the best machine learning-based system up to date (TaggerOne).

The dimensionality of the input word embeddings of all words are set to 50 (i.e. *t*=50). Each dimension is first randomly sampled from uniform distribution *U*[0.25,0.25], then pre-trained by the word2vec tool, an implementation of the unsupervised word embeddings learning algorithm proposed by Mikolov [19] on a unannotated dataset of PubMed biomedical abstracts with about 219 million words, and finally fine-tuned on each training set. We use two kinds of filters of different sizes (i.e. *c*=2 and 3), and set the number of each kind of filter to 50 (i.e., *p*=50). All model parameters are optimized on each training set using 10-fold cross validation, the dimensionality of the input word embeddings of all words are set to 50 (i.e. *t*=50), the best one choosen from [50,100,200] and set the number of each kind of filter to 50 (i.e., *p*=50), the best one choosen from [10,20,50,100] and the model performance is measure by accuracy (i.e., the proportion of mentions correctly normalized) on each test set.

## Results

Our CNN-based ranking biomedical entity normalization system using morphological information (denoted by “CNN-based ranking” in Table 3) is better than the system without using morphological information (denoted by “CNN-based ranking^#^”), and achieves highest accuracies of 90.30% on the ShARe/CLEF test set and 86.10% on the NCBI test set, respectively, which are much higher than the accuracies achieved by the rule-based baseline system (see Table 3, where NA denotes no result reported). The improvement of accuracies on the two test sets ranges from 0.77 to 1.45% with an average of 0.74%. The best CCN-based ranking system outperforms UWM by 0.8% in accuracy on the ShARe/CLEF test set. Compared with D’Souza & Ng’s system, our best CNN-based ranking system shows much higher accuracy on the NCBI test set, but a little lower accuracy on the ShARe/CLEF test set. Compared with TaggerOne, the accuracy of our best CNN-based ranking system is lower by about 2.70% on the NCBI test set. It should be stated here that our reimplementation of D’Souza & Ng’s system obtains the same accuracy on the NCBI test set, but different accuracy on the ShARe/CLEF test set as we cannot completely reconstruct the dictionary used in their system but not released, which may be the main reason why our system performance is a little lower than their.

**Table 3:** Comparison of our CNN-based ranking biomedical entity normalization system with other systems (accuracy)

	ShARe/CLEF (%)	NCBI (%)
CNN-based ranking	90.30	86.10
CNN-based ranking^#^	90.21	85.53
Baseline	89.53	84.65
D’Souza & Ng’s system	90.75	84.65
UWM	89.50	NA
DNorm	NA	82.20
TaggerOne	NA	88.8

## Discussion

In this paper, we propose a CNN-based ranking method for biomedical entity normalization, which can take advantages of not only semantic information but also morphological information of biomedical entity mentions. Experiments on two benchmark datasets show that the proposed CNN-based ranking method outperforms other state-of-the-art systems that only consider morphological information.

Our CNN-based ranking system uses the same rules as the baseline system to generate candidates of entity mentions, and show much better performance than the baseline system. The reason lies in that the CNN-based ranking system chooses the most semantic similar entity to each entity mention from candidates most morphological similar to the mention instead of choosing a entity by artificially defined priority levels. Semantic information of entity mentions provides a route to handle the two cases mentioned in the “Background” section. For example, entity mentions “tremulousness” and “tremulous” (from the ShARe/CLEF test set) with similar morphology are normalized into “tremors” and “tremulous” by the rule-based baseline system, among which “tremulousness” is wrongly normalized, but they are both correctly normalized into “tremulous” by our CNN-based ranking system. Entity mentions “metaplastic polyps of the colorectum” and “colonic polyps” (from the NCBI test set) with significant different morphology are normalized into “polyps” and “colonic polyps”, among which “metaplastic polyps of the colorectum” is wrongly normalized, but they are correctly normalized “colorectal polyps” and “colonic polyps” by our CNN-based ranking system respectively. The normalization process of the two systems for these four mentions is shown in Table 4 in detail. Although our system does not show better results than some state-of-the-art systems using rich manually-craft features (e.g., TaggerOne), this study proves the potentiality of CNN on biomedical entity normalization. How to integrate the systems using rich manually-crafted features and the CNN-based systems together for further improvement may be a direction for future work.

**Table 4:** Normalization process of the rule-based baseline system and our CNN-based ranking system for some entity mentions

Mention	Baseline system	CNN-based ranking system
Tremulousness	Normalized into ‘tremors’ by sieve 7.	Normalized into ‘trembles’ by ranking entities in E=E2={tremors, tremulous, neonatal tremor, NIL } with scores {0.9311, 0.9652, 0.0191, 0.0526}.
Tremulous	Normalized into ‘tremulous’ by sieve 1.	Normalized into ‘tremulous’ by ranking entities in E=E2={tremulous } with scores {1.0}.
Metaplastic polyps of the colorectum	Normalized into ‘polyps’ by sieve 10.	Normalized into ‘colorectal polyps’ by ranking entities in E=E3={adenomatous polyps in the colon, colonic polyps, adenomatous polyps of the colon and rectum, colorectal polyps, polyps, adenomatous polyps } with scores {0.0088, 0.0079, 0.0133, 0.0656, 0.0077, 0.0010}.
Colonic polyps	Normalized into ‘colorectal’ by sieve 1.	Normalized into ‘colonic polyps’ by ranking entities in E=E3={ colonic polyps } with scores {1.0}.

There are two main limitations of our CNN-based ranking system although it achieves good performance. Firstly, candidate generation relies on handcrafted rules, which determines the upper boundary of the CNN-based ranking system, that is the proportion of correct entities in all candidate sets. On the ShARe/CLEF and NCBI test sets, the upper boundaries are 91.33 and 87.45% respectively, indicating that there are a number of candidates beyond the rules. Secondly, the CNN-based ranking system completely ignores ambiguity of entity mentions. To evaluate the effect of ambiguity, we calculate the proportion of ambiguous entity mentions in all entity mentions, which is 4.48% on the ShARe/CLEF test set and 1.56% on the NCBI test set. Among these ambiguous mentions, 25.83% on the ShARe/CLEF test set and 13.33% on the NCBI test set are wrongly normalized. Besides ambiguity errors, there are also many other errors in our system: 1) mapping errors between various entity mentions such as “downbeat nystagmus” that should be mapped to “symptomatic nystagmus”, but wrongly mapped to “nystagmus, myoclonic”; 2) mappings errors caused by “NIL” such as “cerebral microbleeds” that should be mapped to “NIL”, but worngly mapped to “brain ischemia” and “oscillopsia” that should be mapped to “cyclophoria”, but wrongly mapped to “NIL”. These two types of errors account for 4.98 and 3.55% on the ShARe/CLEF test set and 12.96 and 0.73% on the NCBI test set of all entity mentions.

For further improvement, there are two possible directions: 1) increase the upper boundary of the CNN-based ranking system through generating some candidates semantic similar to entity mentions; 2) design an additional disambiguation module.

## Conclusions

In this paper, we propose a CNN-based ranking approach for biomedical entity normalization. This approach achieves accuracies of 90.30 and 86.10% when evaluated on the ShARe/CLEF and NCBI datasets, respectively, which are much higher than that of the state-of-the-art rule-based baseline system. Comparison results show that semantic information is beneficial to biomedical entity normalization and can be well combined with morphological information in our CNN architecture for further improvement.

## Abbreviations

ADA-SCID: Adenosine deaminase deficiency
CLEF: Conference and labs of the evaluation forum
CNN: Convolutional neural network
NCBI: National center for biotechnology information
ShARe: Shared annotated resources
UMLS: Unified medical language system
X-SCID: X-linked combined immunodeficiency diseases
